# Age-related hypertrophy of adenoid and tonsil with its relationship with craniofacial morphology

**DOI:** 10.1186/s12887-023-03979-2

**Published:** 2023-04-06

**Authors:** Xin Huang, Xu Gong, Xuemei Gao

**Affiliations:** Department of Orthodontics, Peking University School and Hospital of Stomatology, 22 Zhongguancun South Avenue, Haidian District, Beijing, 100081 P.R. China

**Keywords:** Adenoid, Tonsil, Hypertrophy, Craniofacial morphology, Subtype

## Abstract

**Background:**

When analyzing the relationship between adenotonsillar hypertrophy and craniofacial morphology, researchers generally regarded hypertrophied adenoids and tonsils as a whole. It remains unclear whether different enlarged sites of pharyngeal lymphoid tissue would correlate with multiple craniofacial subtypes. We hypothesized there would be craniofacial subtypes correlated with different locations of hypertrophied adenoid and tonsil.

**Methods:**

Lateral cephalometric radiographs were obtained from 466 children (171 boys and 295 girls, aged 12.27 ± 2.69 years). They were divided into four groups according to different sites of enlarged pharyngeal lymphoid tissue: adenoid hypertrophy group (AG, n = 126), tonsillar hypertrophy group (TG, n = 59), adenotonsillar hypertrophy group (ATG, n = 69) and control group (CG, n = 212). Five commonly used angles for cephalometric measurements were investigated: SNA (Sella-Nasion-Point A), SNB (Sella-Nasion-Point B), ANB (Point A-Nasion-Point B), mandibular plane angle (MP/SN) and Y-axis angle (SGn/FH).

**Results:**

Children with isolated tonsillar hypertrophy correlated with increased SNA (unstandardized regression coefficient B = 1.38, *p* = 0.009) and SNB (B = 1.99, *p* = 0.001) compared with controls. However, children with isolated adenoid hypertrophy correlated with decreased SNB (B=-0.94, *p* = 0.036), increased ANB (B = 0.74, *p* = 0.014) and increased MP/SN (B = 2.22, *p* < 0.001). Similarly, children with adenotonsillar hypertrophy correlated with decreased SNB (B=-1.36, *p* = 0.015), increased ANB (B = 1.35, *p* < 0.001) and increased MP/SN (B = 2.64, *p* = 0.001).

**Conclusions:**

Isolated adenoid hypertrophy correlated with a retrognathic mandible, an increased maxillo-mandibular sagittal discrepancy, and an increased mandibular plane angle. Isolated tonsillar hypertrophy correlated with maxillary and mandibular protrusion. Adenotonsillar hypertrophy did not show a superimposed craniofacial pattern of the above two but showed the same craniofacial pattern as isolated adenoid hypertrophy.

## Background

Adenoid and tonsil tissue constitute most of Waldeyer’s ring [1]. Increased upper airway resistance related to adenotonsillar hypertrophy is the main pathogenetic abnormality in children with obstructive sleep-disordered breathing (SDB) [2]. The relationship between SDB and craniofacial morphology has been a hot topic and has been extensively studied in decades [3–16]. However, when analyzing the relationship between adenotonsillar hypertrophy and craniofacial morphology, previous studies generally ignored the different locations of adenoids and tonsils. Few studies carefully compared the craniofacial characteristics of children with different locations of enlarged pharyngeal lymphoid tissue which might be potential confounding factors [5, 17–19].

Linder-Aronson [20] first reported that mouth breathing caused by adenoid hypertrophy produced “adenoid facies” which was characterized by an increased anterior facial height, a steep mandibular plane angle, and a retrognathic mandible when compared with healthy controls. In later researches, Trotman [17] found that there were two subtypes of craniofacial morphology. One was adenoid hypertrophy which was characterized by an en bloc backward rotation of the maxilla and mandible relative to the cranial base and by an increased mandibular plane angle. The other was tonsillar hypertrophy which was characterized by a forward relocation of the maxilla and mandible relative to the cranial base and by a decreased mandibular plane angle. However, Behlfelt [3] brought up the controversary that the craniofacial morphology in children with tonsillar hypertrophy were similar to those children with adenoid hypertrophy. Moreover, as for children with not only adenoid hypertrophy but also tonsillar hypertrophy, it was reported that their craniofacial morphology was somewhere between adenoid hypertrophy and tonsillar hypertrophy [18, 19]. Thus, due to the potential confounding factors of different locations of enlarged pharyngeal lymphoid tissue, the results of previous research were inconsistent and conflicting. The craniofacial morphology of children with different locations of enlarged pharyngeal lymphoid tissue is worth further investigation.

Based on the above research reports, we proposed that there would be craniofacial subtypes correlated with different locations of adenoid and tonsillar hypertrophy. We also hypothesized that adenotonsillar hypertrophy might have a superimposed craniofacial pattern of adenoid hypertrophy and tonsillar hypertrophy to reach an intermediate state.

## Materials and methods

This was a retrospective study. This study was performed in line with the principles of the Declaration of Helsinki. Approval was granted by the Ethics Committee of Peking University School and Hospital of Stomatology (Issuing number: PKUSSIRB-202,054,046).

### Subjects

The sample population was taken from consecutive orthodontic patients of the researchers from June 2014 to August 2019. Inclusion criteria were those children between 3 and 18 years old with complete medical records and cephalograms. Exclusion criteria were as follows: previous orthodontic treatment, history of tonsillectomy or adenoidectomy, obesity according to the norm of Chinese children and adolescents [21], history of craniofacial injury, syndrome or congenital abnormality, cases of severe skeletal abnormalities requiring surgical treatment, with systemic disorders or neurological diseases, and with upper airway obstruction related diseases, such as nasal polyps, nasal tumor, nasal deformity, severe deviation of nasal septum, severe turbinate hypertrophy and severe allergic rhinitis.

In the end, 466 Chinese children (171 boys and 295 girls) from 531 Chinese children aged 3 to 18 years were included in this study. All children had routine cephalometric examinations before treatments. Adenoid hypertrophy and tonsillar hypertrophy were assessed by lateral cephalometric analysis. Based on the cephalometric analysis, children were divided into four groups according to the locations of the pharyngeal lymphoid tissue enlargement. Adenoid hypertrophy group (AG) consisted of 126 children with the adenoid more than half of the airway diameter and the tonsil less than half of the airway diameter. Tonsillar hypertrophy group (TG) consisted of 59 children with the adenoid less than half of the airway diameter and the tonsil more than half of the airway diameter. Adenotonsillar hypertrophy group (ATG) consisted of 69 children with both the adenoid and the tonsil more than half of the airway diameter. Control group (CG) consisted of 212 children with both the adenoid and the tonsil less than half of the airway diameter. Details of demographic information of four groups are shown in Table 1.

**Table 1:** Demographic characteristics and statistical comparisons of patients in adenoid hypertrophy group, tonsillar hypertrophy group, adenotonsillar hypertrophy group and control group

Variables	AG (n = 126)	TG (n = 59)	ATG (n = 69)	CG (n = 212)	*P* value	Multiple comparison					
						AG-TG	AG-ATG	AG-CG	TG-ATG	TG-CG	ATG-CG
Age, y^a^	11.73 ± 2.51	12.47 ± 2.72	11.07 ± 3.35	12.92 ± 2.34	< 0.001***	0.290	0.485	< 0.001***	0.049*	0.663	< 0.001***
Gender, male(%)^b^	56(44.4%)	21(35.6%)	33(47.8%)	61(28.8%)	0.005**	0.255	0.650	0.003**	0.162	0.313	0.004**
BMI (kg/m^2^)	18.67 ± 3.47	18.31 ± 2.84	17.54 ± 4.24	18.08 ± 2.80	0.386	-	-	-	-	-	-

Values are expressed as means ± standard deviations or frequencies (percentages)

AG, adenoid hypertrophy group, TG, tonsillar hypertrophy group, ATG, adenotonsillar hypertrophy group, CG, control Group, BMI, body mass index

^a^ Welch’s test followed by Games-Howell’s test

^b^ Chi-square followed by partitioning chi-square

* *P* < 0.05, ** *P* < 0.01, *** *P* < 0.001

### Cephalometric analysis

**Fig. 1:**
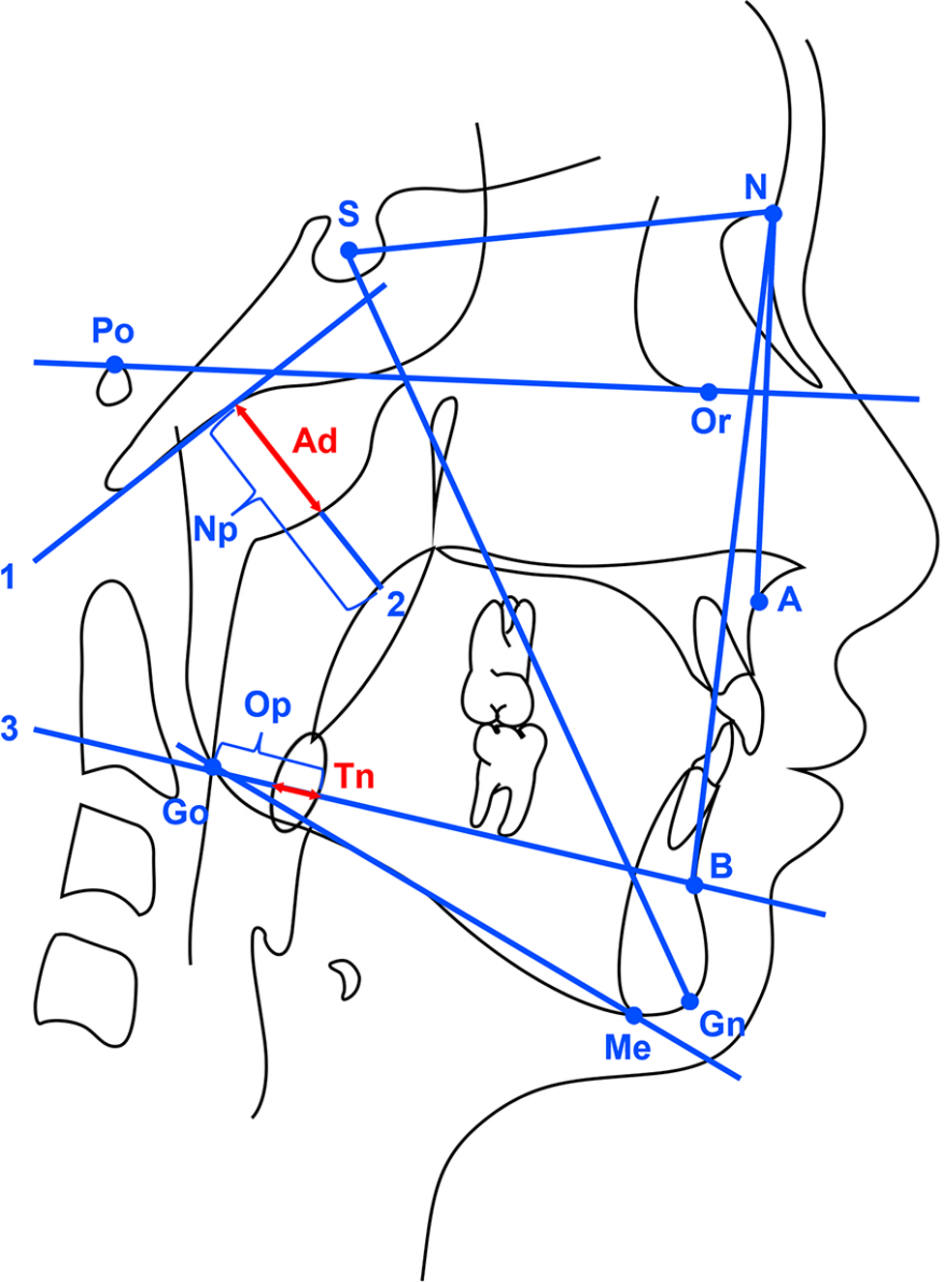
Cephalogram illustrating the cephalometric landmarks and measurements of adenoid and tonsil **S**, sella; **N**, nasion; **A**, subspinale; **B**, supramentale; **Go**, gonion; **Me**, menton; **Gn**, gnathion; **Po**, Porion; **Or**, Orbitale; **Line 1**, the line parallel to anterior margin of basiocciput; **Line 2**; the line perpendicular to line1 at the most convex point of the adenoid shadow, where the width of the adenoid and nasopharynx is measured; **Ad**, the width of adenoid measured on line 2; **Np**, the width of nasopharynx measured on line 2; **Line 3**, the line between point B and point Go, where the width of the tonsil and oropharynx is measured; **Tn**, the width of the tonsil on line 3; **Op**, the width of oropharynx on line 3; **Ad/Np**, the size of adenoid; **Tn/Op**, the size of tonsil

All children had routine cephalometric examinations before treatment, which were performed by radiology specialists using orthopantomograph OC200 digital x-ray machine (Instrumentarium Dental, Tuusula, Finland). The lateral cephalograms were taken with children in an upright position and the Frankfort horizontal parallel to the floor. All children were instructed to remain still and to maintain centric occlusion without moving head or making speech or swallowing. Cephalometric analysis was performed by a single investigator. Craniofacial and pharyngeal lymphoid tissue measurements were generated by selecting landmarks through Huazheng software (https://www.huazhengcl.com/). Cephalometric landmarks and measurements in this study are shown in Fig. 1. The landmarks included sella (S), nasion (N), subspinale (A), supramental (B), menton (Me), gonion (Go) and gnathion (Gn). Frankfort Horizontal (FH) plane is one of the most widely used intracranial landmarks, defined by the line passing through the orbitale (Or) and porion (Po). Mandibular plane (MP) constructed using the points menton (Me) and gonion (Go). SNA angle describes the anteroposterior projection of the maxilla. SNB angle describes the anteroposterior projection of the mandible. ANB angle is equal to SNA angle minus SNB angle, which describes the anteroposterior intermaxillary relationship. MP/SN angle describes mandibular divergence. SGn/FH angle describes the direction of growth. The size of adenoid was determined as the proportion of adenoid (Ad) to nasopharynx (Np). The size of tonsil was determined as the proportion of tonsil (Tn) to oropharynx (Op). The criteria for the analysis of adenoid hypertrophy and tonsillar hypertrophy from the lateral cephalograms were referred to the study by Baroni et al. [18]. Ad/Np ratio more than 0.5 was considered to constitute adenoid hypertrophy. Tn/Op ratio more than 0.5 was considered to constitute tonsillar hypertrophy. According to the individual dentition stage, children were assigned to three sagittal skeletal patterns based on the cephalometric norm of Chinese children [22]: skeletal class I (3.3°≤ANB ≤ 6.1° in mixed dentition, 0.7°≤ANB ≤ 4.7° in permanent dentition), skeletal class II(ANB > 6.1° in mixed dentition, ANB > 4.7° in permanent dentition) and skeletal class III(ANB < 3.3° in mixed dentition, ANB < 0.7° in permanent dentition). To evaluate the error of the method, 20 lateral cephalograms selected randomly were re-traced and re-measured after 2 weeks by the same investigator. The intraclass correlation coefficients (ICCs) varied between 0.88 and 0.94 for the cephalometric measurements, indicating a satisfactory level of intra-investigator reliability.

### Statistics

The normality of the distribution of continuous variables were checked by Shapiro-Wilk test. Levene´s test was used to examine the homogeneity of variance. All the continuous variables approached a normal distribution but showed a heteroscedasticity. Thus, the continuous variables were expressed with means ± standard deviations. Welch’s test followed by Games-Howell´s test was used to detect statistically significant differences between groups. Categorical variables were expressed as frequencies and percentages. Chi-square for intergroup comparisons and partitioning chi-square for multiple comparisons (*p* < 0.05/the number of comparisons) were conducted. To detect craniofacial differences between groups, multiple linear regression analysis was performed, using every single cephalometric variable as a dependent variable and age, sex, and the groups (converted to dummy variables) as independent variables. Unstandardized regression coefficients B were calculated, representing age- and sex-adjusted differences between groups. Then, when sample size was limited to AG and TG, stepwise multiple regression was conducted to find the variables that best differentiated adenoid hypertrophy and tonsillar hypertrophy in terms of craniofacial characteristics. Unless otherwise stated, *p* < 0.05 was regarded as indicating statistical significance. Statistical analyses were performed using IBM SPSS Statistics for Mac (Version 26.0. Armonk, NY: IBM Corp.) The histograms and line charts were drawn using GraphPad Prism 8. Schematic diagram of cephalometric were created with Adobe Photoshop 2020.

## Results

Due to the large age range, this study did not directly compare the physical measurements, but was analyzed by the proportions of skeletal patterns in different groups and by multiple regression models to avoid the interference of stages of development.

### Demographic characteristics of the subjects

A total of 466 subjects were included in the study (Table 1). Of all the subjects, 126 (27.0%) had isolated adenoid hypertrophy, 59 (12.7%) had isolated tonsillar hypertrophy, 69 (14.8%) had adenotonsillar hypertrophy. Age was normally distributed in each group, however AG and ATG exhibited a younger age than CG. There was a predominance of girls in CG while a relative high proportion of boys in AG and ATG. Body mass index did not differ within groups (*p* = 0.386). Thus, age and sex might be confounding variables which need to be controlled to reach a demographical equivalence. In the following analysis, we used multiple linear regression analysis to control for age and sex differences between groups.

### Age-related changes in adenoid and tonsil

**Fig. 2:**
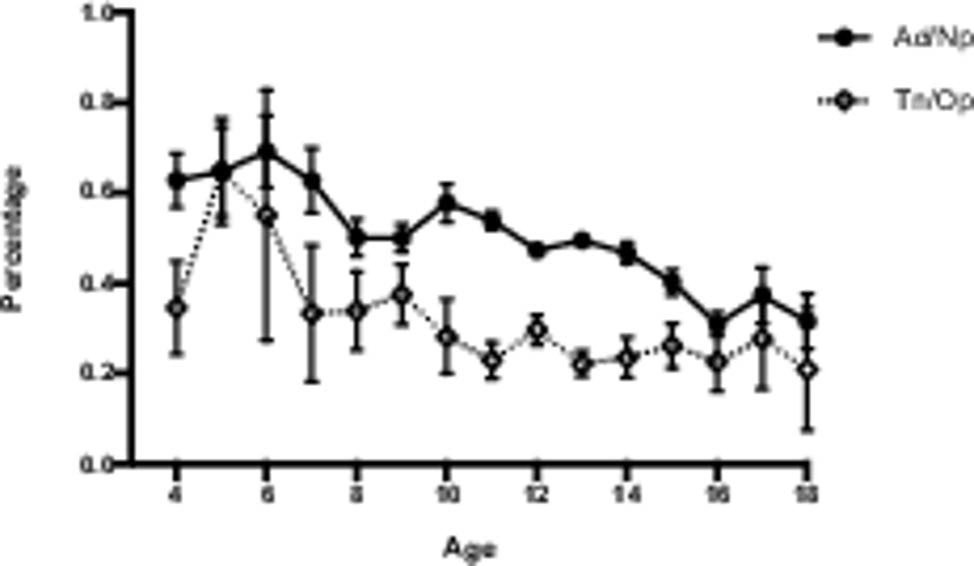
Age-related changes in adenoids and tonsils Ad/Np, the size of adenoid; Tn/Op, the size of tonsil. Dots and error bars represent mean ± standard error

The result of the present study showed that there was an age-related decrease in the size of adenoids and tonsils (Fig. 2). The average proportions of the adenoid and tonsil sizes to the upper airway at various ages were different. The mean of Ad/Np reached maximum (Ad/Np = 0.69 ± 0.08) at 6 years of age, after which it decreased. There was a slight increase in the size of adenoid (Ad/Np = 0.58 ± 0.04) at 10 years of age, followed by a progressive decrease. As for tonsils, the mean of Tn/Op reached maximum (Tn/Op = 0.65 ± 0.20) at 5 years of age, after which the tonsil decreased remarkably and then stayed at a relative low size. The mean of Ad/Np was greater than 0.5 before the age of 12 while Tn/Op started to have a mean less than 0.5 from the age of 7. Thus, adenoids occupied a larger proportion of the corresponding airway for a longer period of time compared with tonsils.

### Associations between cephalometric variables and different obstructive sites of upper airway

**Fig. 3:**
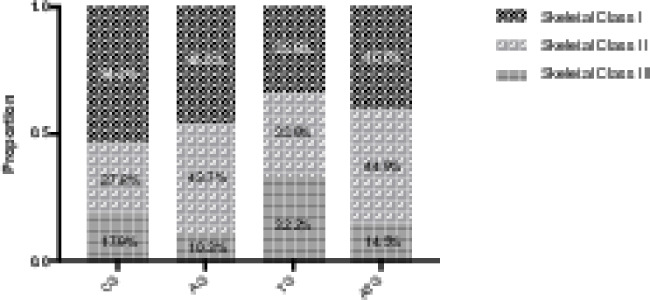
Different sagittal skeletal patterns in adenoid hypertrophy group, tonsillar hypertrophy group, adenotonsillar hypertrophy group and control group AG, adenoid hypertrophy group; TG, tonsillar hypertrophy group; ATG, adenotonsillar hypertrophy group; CG, control Group

The proportions of sagittal skeletal patterns in CG, AG, TG and ATG were examined (Fig. 3). Chi-square test showed that the proportions of sagittal skeletal patterns in different groups were statistically different (*p* < 0.001). Compared with CG (skeletal class II = 27.8%), the proportions of skeletal class II in AG (43.7%, *p* = 0.013) and ATG (44.9%, *p* = 0.011) were significantly increased. Compared with CG (skeletal class III = 17.9%), the proportion of skeletal class III in TG (32.2%, *p* = 0.004) was significantly increased.

**Fig. 4:**
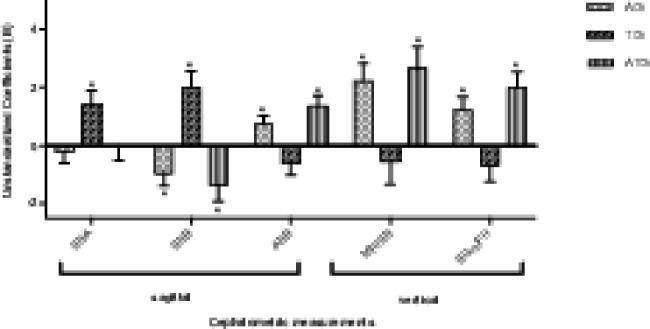
Comparisons of multiple linear regression analysis of cephalometric variables AG, adenoid hypertrophy group; TG, tonsillar hypertrophy group; ATG, adenotonsillar hypertrophy group. The zero line represents the control group, and the difference between each angle measurements and the control are displayed as histograms. Asterisk represents statistically significant

Multiple linear regression (Table 2; Fig. 4) showed that TG was positively correlated with SNA (unstandardized regression coefficient B = 1.38, *p* = 0.009) and SNB (B = 1.99, *p* = 0.001) when compared with CG. In other words, isolated tonsillar hypertrophy correlated with maxillary and mandibular protrusion compared with controls. However, AG was positively correlated with ANB (B = 0.74, *p* = 0.014), MP/SN (B = 2.22, *p* < 0.001) and FH/SGn (B = 1.22, *p* = 0.006) but negatively correlated with SNB (B = -0.94, *p* = 0.036) when compared with CG. In other words, isolated adenoid hypertrophy correlated with a retrognathic mandible, an increased maxillo-mandibular sagittal discrepancy, and an increased mandibular plane angle. Similarly, ATG was positively correlated with ANB (B = 1.35, *p* < 0.001), MP/SN (B = 2.64, *p* = 0.001) and FH/SGn (B = 1.99, *p* < 0.001) but negatively correlated with SNB (B = -1.36, *p* = 0.015) when compared with CG. In other words, adenotonsillar hypertrophy represent a retrognathic mandible, an increased maxillo-mandibular sagittal discrepancy, and an increased mandibular plane angle, which was similar with isolated adenoid hypertrophy.

**Table 2:** Multiple linear regression analysis of cephalometric variables in adenoid hypertrophy group, tonsillar hypertrophy group, adenotonsillar hypertrophy group and control group

Dependent variable		AG^a^		TG^a^		ATG^a^	
		B value	*P* value	B value	*P* value	B value	*P* value
Sagittal	SNA(°)	-0.20	0.620	1.38	0.009**	-0.01	0.992
	SNB(°)	-0.94	0.036*	1.99	0.001**	-1.36	0.015*
	ANB(°)	0.74	0.014*	-0.61	0.114	1.35	< 0.001***
Vertical	MP/SN(°)	2.22	< 0.001***	-0.55	0.487	2.64	0.001**
	FH/SGn (°)	1.22	0.006**	-0.68	0.231	1.99	< 0.001***

AG, adenoid hypertrophy group, TG, tonsillar hypertrophy group, ATG, adenotonsillar hypertrophy group

B value: unstandardized regression coefficients representing age- and sex-adjusted differences between groups

^a^ Reference group: control group

* *P* < 0.05, ** *P* < 0.01, *** *P* < 0.001

When sample size was limited to AG and TG, stepwise multiple regression analysis tested the cephalometric variables which were significantly correlated to the size of adenoids and tonsils (Table 3). The results showed that SNB was the only significant cephalometric variable. Adenoid hypertrophy correlated with a decreased SNB (B = -0.01, *p* < 0.001) and tonsillar hypertrophy correlated with an increased SNB (B = 0.02, *p* < 0.001).

**Table 3:** Stepwise multiple regression analysis of Ad/Np and Tn/Op on cephalometric variables

Stepwise multiple regression with Ad/Np as dependent variable					
	Unstandardized Coefficients B	Standard error	95% Confidence interval	*P* value	Adjusted R^2^
Constant	1.50	0.196	1.11,1.89	< 0.001***	0.131
SNB	-0.01	0.003	-0.02,-0.01	< 0.001***	
**Stepwise multiple regression with Tn/Op as dependent variable**					
	Unstandardized Coefficients B	Standard error	95% Confidence interval	*P* value	Adjusted R^2^
Constant	-1.21	0.429	-2.05,-0.36	0.005**	0.052
SNB	0.02	0.006	0.01,0.03	0.001**	

Ad/Np, the size of adenoid; Tn/Op, the size of tonsil

Regression analyses were performed in 185 patients with isolated adenoid hypertrophy or isolated tonsillar hypertrophy. Patients with adenotonsillar hypertrophy has been excluded. Age and sex have been adjusted. Only significant data are presented

* *P* < 0.05, ** *P* < 0.01, *** *P* < 0.001

## Discussion

The present study found that identifying the specific obstructive sites of the upper airway is important because different combinations of enlarged pharyngeal lymphoid tissue corresponded to different craniofacial subtypes.

In the present study, we found that growing pharyngeal lymphoid tissue narrowed the upper airway to variable degrees in early childhood and the degree of airway obstruction decreased with age. Even though both adenoids and tonsils belong to Waldeyer’s ring, they had different growth patterns, which suggesting that we should pay attention to the difference between adenoids and tonsils.

In the present study, isolated adenoid hypertrophy correlated with a retrognathic mandible, an increased maxillo-mandibular sagittal discrepancy, and an increased mandibular plane angle. While isolated tonsillar hypertrophy correlated with maxillary and mandibular protrusion. By stepwise regression, we found that SNB was a significant variable which could differentiate craniofacial characteristics of adenoid hypertrophy and tonsillar hypertrophy.

One possible explanation is based on Moss’s theory of functional matrix, which indicating that craniofacial growth is the result of both genetic and functional factors [23]. Adenotonsillar hypertrophy could cause upper airway narrowing in children. When upper airway resistance increases, mouth-breathing often occurs, leading to postural and functional alterations in the oro-facial system in order to search for a more efficient airflow against obstructed upper airway [24–27]. The imbalance of different tissues within the ‘matrix’ of the oro-facial capsule affects the growth and development of craniofacial and dentofacial structures [23]. For example, when tonsils get enlarged, the tongue will be forced to be postured forward [4, 17]. Tongue would act as a stimulation factor to activate forward growth of the mandible [5]. On the contrary, adenoid hypertrophy results in downward position of the tongue and the mandible and extended head posture [6, 20], which further leads to a retrognathic mandible and a steep mandible angle plane [7, 12, 16]. Another hypothesis that must be mentioned is that continuous airflow through the nasal cavity produces a constant stimulation for the lateral growth of maxilla and for the lowering of the palatal vault [28, 29]. However, nasal obstruction resulted from adenoid hypertrophy could cause the absence of negative pressure in the nasal cavity, which prevents the lowering of the palatal vault. Together with the open mouth posture, the muscles exert inward pressure on the maxillary and maxillary arches, which results in high arched palates. As a result, the growth of maxilla was limited transversally and more pronounced vertically.

As for adenotonsillar hypertrophy, previous studies found that the craniofacial morphology of children with adenotonsillar hypertrophy was somewhere between adenoid hypertrophy and tonsillar hypertrophy [18, 19]. However, we did not find a superimposed craniofacial pattern in children with adenotonsillar hypertrophy, which rejected our null hypothesis. Instead, we found that both children with isolated adenoid hypertrophy and children with adenotonsillar hypertrophy correlated with a retrognathic mandible, an increased maxillo-mandibular sagittal discrepancy, and an increased mandibular plane angle. Moreover, compared with isolated adenoid hypertrophy, children with adenotonsillar hypertrophy tended to have a more severe discrepancy despite it was not significant. We could speculate that the adenoids and tonsils act as a synergy. This result concurred with the study of Nunes et al. [31]who analyzed the relationship of dental occlusion and obstructive sites of upper airway [30]. Nunes et al. evaluated the dental occlusions of 114 children ranging in age from 3 to 12 years and they found that the rate of class II relationship in children with combined adenoid and tonsil enlargement was higher than that in children with isolated adenoid hypertrophy [31] [30].

Hypertrophy of adenoid and tonsil was normal in early childhood and probably was an index of immunological activity [32] [31]. Growing adenotonsillar tissue narrowed the upper airway to variable degrees in early childhood and the degree of airway obstruction decreased with age, which was supported by another MRI study [33] [32]. The adenoid reached peak at age 6 and showed small increases at age 10 (possibly associated with the sex hormones at puberty), which was consistent with the Linder-Aronson’s longitudinal study [34] [33]. The tonsil reached peak at 5 years of age, which was supported by Shintani’s findings [35] [34]. Besides, we found that adenoids occupied a larger proportion of the corresponding airway for a longer period of time compared with tonsils. Thus, it is possible that adenoids might play a greater role in craniofacial morphology.

The adenotonsillar hypertrophy is not an overwhelming factor and is not the only factor associated with craniofacial morphology. Craniofacial morphology is also correlated with family heredity, personal habits, self-adaptability and so on. Subtypes mentioned above only represented a general trend as a whole. Even though one group as a whole had certain craniofacial features, every individual’s growth and development still varied. For example, in our study, isolated tonsillar hypertrophy group had the highest percentage of skeletal class III but there was still a certain percentage of class I and class II.

The present study could not avoid limitations. First, since it was a retrospective study, there might be some inadequacies in documentations and special condition controlling. For example, respiratory control during cephalometric scanning might have some impact on upper airway [36] [35]. Second, this study was based on lateral cephalograms. Our study sample did not undergo otolaryngology examinations such as flexible nasoendoscopy for adenoids and direct oropharyngeal visual examination for tonsils. Future prospective studies should be carried out by combining otolaryngology examinations, cone beam computer tomography (CBCT) and upper airway resistance and ventilation function evaluations. Third, influenced by ethical aspects, although we had excluded syndromes, congenital malformation, and severe craniofacial deformity and so on, samples from children in orthodontic clinic were not a fair representation of the healthy population. Last, this study was unable to establish a causal relationship between craniofacial morphology and adenotonsillar hypertrophy.

## Conclusions

Although with individual differences in craniofacial morphology, different combinations of enlarged pharyngeal lymphoid tissue corresponded to different craniofacial subtypes as a population trend in children. Isolated tonsillar hypertrophy correlated with maxillary and mandibular protrusion. Isolated adenoid hypertrophy and adenotonsillar hypertrophy correlated with a retrognathic mandible, an increased maxillo-mandibular sagittal discrepancy, and an increased mandibular plane angle. Therefore, in clinical practice, the size of adenoids and tonsils should be evaluated separately for children with “adenoid facies”. Meanwhile, we need to design a personalized treatment plan based on the craniofacial growth trend and family genetic characteristics. More attention should be paid to the mechanism of hypertrophy of adenoids and tonsils in the future.

## Data Availability

The data used in the current study are available from the corresponding author.

## List of Abbreviations

SDB: Sleep-disordered breathing
AG: Adenoid hypertrophy group
TG: Tonsillar hypertrophy group
ATG: Adenotonsillar hypertrophy group
CG: Control group
Ad: Adenoid
Np: Nasopharyngeal
Tn: Tonsil
Op: Oropharyngeal
